# Immune reconstitution inflammatory syndrome in AIDS-related non-hodgkin‘s lymphoma

**DOI:** 10.4103/0971-5851.65346

**Published:** 2009

**Authors:** Uday A. Phatak

**Affiliations:** *Department of Medicine, Shri Siddhivinayak Ganapati Cancer Hospital, Miraj, India*

**Keywords:** *AIDS-related lymphoma*, *HIV-IRIS*, *IRIS*

## Abstract

Immune Reconstitution syndrome following antiretroviral therapy is common in HIV/AIDS patients due to boosting of immunity. A case is reported here wherein AIDS-related Non-Hodgkin‘s lymphoma patient received CHOP regimen and antiretroviral therapy. Patient developed tubercular lymphadenopathy paradoxically as a manifestation of IRIS.

## INTRODUCTION

Immune Reconstitution Inflammatory Syndrome (IRIS) is defined as a paradoxical worsening of the pre-existing opportunistic infection(s) when Human *Immunodeficiency Virus* (HIV) patients are on Highly Active Anti-Retroviral Therapy, and there is significant improvement in the CD4+ T cell count, reduction in viral load, and significant clinical improvement. This paradoxical response is seen mainly in advanced HIV infection (CD4+ T cell count < 50 cells/l). IRIS may manifest as the reactivation of dormant infections, such as, tuberculosis, cryptococcosis or leprosy.[1] Sometimes, malignancies such as Kaposi‘s sarcoma, Non-Hodgkin Lymphoma, cervical or bronchogenic carcinomas may manifest as clinical features of IRIS.[2,3] Their incidence varies from 10 to 37% in different clinical studies and with the types of clinical conditions manifesting as IRIS.[1,4,5] There are hardly any reports on PubMed regarding the Immune Reconstitution Inflammatory Syndrome in *Acquired Immune Deficiency Syndrome* AIDS-related lymphoma following chemotherapy and Highly Active Antiretroviral Therapy (HAART).

A case of AIDS-related Non-Hodgkin Lymphoma (ARL), which was treated with the cyclophosphamide, hydroxydaunorubicin (doxorubicin), Oncovin (vincristine), and prednisone / prednisolone (CHOP) regime and Efavirenz-based Anti-Retroviral Therapy, developed IRIS with tubercular infection within nine months of therapy, and is discussed here.

## CASE REPORT

The 53-year-old male patient presented in April 2003, with fever, right axillary lymphadenopathy, weight loss, and oral candidiasis for two months. Routine investigations confirmed diffuse large B cell lymphoma and HIV-1 infection. Profound immune deficiency was observed. His CD4+ T cell count was 43 cells/L, CD8+ T cell count 339 cells/l and plasma HIV RNA level was 110,174 copies/ml (by PCR ultrasensitive). The physical examination revealed oral thrush, axillary lymphadenopathy, and hepatosplenomegaly. Routine investigations revealed hemoglobin 11.6 Gm/dl, with normal WBC, platelet counts and indices. The bone marrow examination was normal. Liver function tests and renal function tests were normal. S. LDH was raised, (900 IU/L). He was treated with six courses of chemotherapy with CHOP regime. Zidovudine, Lamivudine, and Efavirenz were started for control of HIV infection. He showed significant improvement with chemotherapy and tolerated HAART. He received cotrimaxazole, antifungal, and tuberculosis prophylaxis as well. He was in good condition during the next six months of observation. His CD4+ T lymphocyte count increased significantly and reached 156 cells/L, CD8+ T cell count was 875 cells/l and plasma HIV RNA was less than the level of assay quantification by PCR ultrasensitive (50 copies/ml). [Table 1] There were no signs or symptoms of any disease and the antiretroviral therapy was continued.

**Table 1:** Study of viral load and T cell subpopulations before and after highly active anti-retroviral therapy

Year	Viral load (copies/ml)	CD4 count (cell / microliter)	CD8 count (cell / microliter)	CD4/CD8 ratio
April 2003	110, 154	43	339	0.12
January 2004	< 50	215	875	0.26

Three months later the patient was admitted with painful right axillary lymphadenopathy, fever and sweating on follow-up. An axillary lymph node biopsy demonstrated acid fast bacilli but no evidence of Non-Hodgkin Lymphoma. IRIS with tuberculosis was diagnosed. Routine blood counts, urine analysis, liver function tests, renal function tests and serum electrolytes were normal. There was no evidence of active tuberculosis on chest X-ray. Ultrasonic study of the abdomen and pelvis showed hepatosplenomegaly. The antituberculosis regimen and corticosteroids along with HAART were started for tubercular lymphadenopathy. He developed hyperlipidemia following anti-Retroviral therapy. Serum cholesterol was 355 mg per deciliter. He was prescribed Atorvastatin 20-mg daily along with anti-Retroviral, antitubercular agents. After six years, the patient is receiving regular HAART and is in good health.

## DISCUSSION

Real incidence of AIDS-related lymphoma in India is not known. Non-Hodgkin Lymphoma (NHL) is the most common AIDS-defining cancer in Indian patients. In an Indian study of 250 cases of AIDS-associated cancers, 30.8% had Non-Hodgkin Lymphoma,[6] while in a US study involving 441 cases of AIDS-associated cancers, 51.5% had Kaposi sarcoma and 18.6% had NHL.[7] Combination chemotherapy along with anti-retroviral therapy has been considered the standard treatment for AIDS-related lymphomas. There was 32% without HAART and 57% with HAART. Longevity was significantly improved in the latter group. Clinical outcome in patients with AIDS-related Non-Hodgkin‘s Lymphoma, particularly diffuse large B-cell lymphoma, taking HAART, was almost similar to that of *de novo* lymphoma.[8] However, there are no definite guidelines with regard to antiretroviral therapy for AIDS-related lymphomas.

Protease-inhibitor based Highly Active Anti-Retroviral Therapy is expensive and is associated with profound neutropenia when concurrent chemotherapy is administered and alterations in anticancer drug levels, due to drug–drug interactions.[9] When HAART is combined with the CHOP regimen, pharmacokinetics of all agents except Doxorubin are altered with protease inhibitors such as, Saquinavir, Nelfinavir, and Indinavir.[10] Non-PI based regimes, on the contrary, are less expensive, have fewer complications and drug–drug interactions. It is not known, how frequently patients with AIDS-related lymphoma on chemotherapy and Highly Active Anti-Retroviral Therapy develop the Immune Reconstitution Inflammatory Syndrome. Risk factors for development of IRIS include high baseline CD4+ count, ratio of CD4+/CD8+ > 0.15, > 2 log fall in the viral load after 90 days of HAART, and an early initiation of ART after opportunistic infection.[11,12] In this case-report, fall in viral load was significant and the baseline CD4 count was around 200 cells per microliter. IRIS features followed after administration of ART, but were not related to any type of anti-retroviral agents. HIV-associated IRIS developed after a mean of 33 days following cancer treatment, including bone marrow transplantation and correction of neutropenia. The median age was 56 years and overall clinical outcome was favorable.[4]

Corticosteroids such as methylprednisone along with other specific treatments, are required for the control of HIV-associated IRIS.[4] AIDS-associated cancers with Immune Reconstitution Inflammatory Syndrome receiving multiple agents and corticosteroids often have a favorable outcome.
